# A new nodule-associated bacterium*, Cupriavidus* consociatus sp. nov. Isolated from the root nodules of *Leucaena* sp. and *Arachis* sp. growing in a cacao field in Chiapas, Mexico

**DOI:** 10.1371/journal.pone.0324390

**Published:** 2025-05-27

**Authors:** Erika-Yanet Tapia-García, Belén Chávez-Ramírez, Violeta Larios-Serrato, Ivan Arroyo-Herrera, J. Antonio Ibarra, Paulina Estrada-de los Santos

**Affiliations:** 1 Departamento de Microbiología, Escuela Nacional de Ciencias Biológicas, Instituto Politécnico Nacional. Ciudad de México, México; 2 Departamento de Bioquímica, Escuela Nacional de Ciencias Biológicas, Instituto Politécnico Nacional. Ciudad de México, México; Universidade de Coimbra, PORTUGAL

## Abstract

*Cupriavidus* is a genus of bacteria that inhabit diverse ecological niches, including plant-associated and nodulating species. A previous survey of legume plants in the south of Mexico resulted in the isolation of several bacteria. This present study describes two *Cupriavidus* strains isolated from the nodules of *Leucaena* sp. and *Arachis* sp. plants growing in a cacao field in Chiapas, Mexico. Both strains (LEh25^T^ and LEh21) shared identical 16S rRNA gene sequences and 98.4% identity with *Cupriavidus oxalaticus* Ox1^T^. However, the *in silico* average nucleotide identity (ANI) and digital DNA-DNA hybridization (dDDH) values (99.99 and 99.90% similarity, respectively) indicated that they belonged to different genomic species when compared to type strains of *Cupriavidus* species (ANI ~ 93.2 and dDDH ~ 50% similarity). Phylogenomic analysis indicated that the novel species would be placed in the genus *Cupriavidus* next to *C. oxalaticus* Ox1^T^. Neither strain could fix nitrogen in a semisolid medium, and the interactions of the type strain with *Phaseolus vulgaris,* and *Leucaena* sp. revealed the formation of nodules, although these were ineffective. The genomic analysis demonstrated the presence of nitrogen fixation and nodulation genes with the same organization as in other strains of *Cupriavidus* and *Paraburkholderia*, although lacking NodB. To complement the study of the novel species, the strains were phenotypically and chemotaxonomically analyzed, with the results indicating differences with *C. oxalaticus* Ox1^T^ and other similar type strains of *Cupriavidus* species. From these results, we propose the novel species *Cupriavidus consociatus* sp. nov. with the type strain LEh25^T^=TSD-314^T^ = CDBB B-2085^T^.

## Introduction

*Cupriavidus* is a genus of bacteria belonging to the class *Betaproteobacteria*. Currently, the genus contains 23 species, with three names not yet validly published (“*C. eutrophus*”, “*C. malaysiensis*” and “*C. neocaledonicus*”) according to the International Code of Nomenclature of Prokaryotes (ICNP) in the List of Prokaryotic names with Standing in Nomenclature (https://www.bacterio.net/). *Cupriavidus* species have been isolated from clinical specimens, plant rhizosphere samples, soil, water, and legume nodules. The genus occurs worldwide. Some strains of various *Cupriavidus* species are tolerant to heavy metals. *Cupriavidus*, which means lover of copper (*cuprum* = copper and *avidus* = eager for, loving), includes the species *C. metallidurans,* a bacterium with exceptional metal tolerance. This species can grow in the presence of silver, copper, zinc, lead, and other metals [1]. *C. metallidurans* has been proposed for the use in the bioremediation of mercury-polluted agricultural soils [2]. Other strains belonging to different *Cupriavidus* species with resistance to several metals are *C. necator, C. alkaliphilus, C. plantarum, C. agavae, C. nantongensis, C. campinensis, C. pauculus, C. gilardii, C. taiwanensis, C. basilensis,* and *“C. neocaledonicus”* [3–11]. However, an essential characteristic of several species is their involvement in human infections; besides *C. metallidurans,* the species *C. gilardii, C. pauculus, C. respiraculi*, *C. cauae, C. basilensis,* and *C. taiwanensis* occur in clinical settings [12,13]. Another feature of *Cupriavidus* is that several strains belonging to different species are plant-associated bacteria. Members of the class Alphaproteobacteria were once thought to be the only bacteria to nodulate legumes until *C. taiwanensis,* belonging to the Betaproteobacteria, was demonstrated to possess this activity [14,15]. Currently, *Cupriavidus necator* and “*Cupriavidus neocaledonicus*” and many other strains of *Cupriavidus* (not yet officially described) can nodulate legumes, especially plants from the Mimoseae tribe [16]. A recent analysis of legume nodules from the south of Mexico revealed the presence of many bacterial genera, including the genus *Cupriavidus* [17]. In the present study, we employed phenotypic, chemotaxonomic, and genomic analysis of two bacterial strains, identifying them as a novel *Cupriavidus* species with the ability to nodulate legumes but ineffective.

## Materials and methods

### Bacterial strains

*Cupriavidus* strain LEh25^T^ was isolated from nodules of *Arachis* sp. and strain LEh21 was from nodules of *Leucaena* sp.; both legumes were growing in a cacao field in Chiapas, Mexico (N 14° 52’ 30.18” W 92° 21’ 24.768”) [17]. The owner of the cacao field granted permission for the sample collection; therefore, no official documents were required. The bacteria were isolated by sampling five randomly chosen legume nodules selected from five wild plants. The nodules were washed three times with sterile water, then immersed in 100% ethanol at 96° for 30 s, 10% sodium hypochlorite for 10 min, and then rinsed five times with sterile water. The water from the final washing was placed in LB medium plates (BD Bioxon) to verify surface disinfection. The nodules were crushed with a plastic pestle in 40 mL of sterile water. The whole nodule suspensions were inoculated onto plates with yeast extract mannitol (YM) medium containing 5 g/L of mannitol. The plates were incubated at 30°C for 3–5 days. The bacterial isolates were stored in 35% glycerol at -70°C until further analysis.

### 16S rRNA gene sequence analysis

The 16S rRNA gene sequences from strain LEh25^T^ (MN830085) and LEh21 (MN830086) were updated from a previous study, as the latter were too short to obtain appropriate information [17]. The 16S rRNA gene fragments were amplified with primers 27F/1492R [18] and sequenced by Macrogen Inc. (https://dna.macrogen.com). The sequences were edited and assembled with ChromasPro 2.1.5 (Technelysium Pty Ltd) and compared with sequences on the EzBioCloud website to determine the closest species of *Cupriavidus*. A phylogenetic analysis was performed with all type strains of *Cupriavidus* species. The alignment was carried out by Clustal Omega [19]. The aligned sequences were used for the estimation of the evolutionary model with IQ-TREE 2 v 2.3.6 [20]. The phylogeny was obtained using the Bayesian inference method with the Beast software v 2.5 [21], and the GTR + I + G model. A total of 10^7^ generations were performed, after which 25% of the trees were discarded [22]. *Ralstonia solanacearum* LMG 2299^T^ was included as an outgroup. The software PhyML 3.1 was used for tree construction. The phylogenetic tree was displayed with FigTree v 1.4.4 (http://tree.bio.ed.ac.uk/software/figtree/).

### Whole genome sequencing

The strains LEh25^T^ and LEh21 were grown in 40 mL LB broth and incubated overnight (120 rpm) at 30°C. The total DNA was isolated using Moore and Dowhan’s method [23]. The genome sequence was obtained by Novogene (https://en.novogene.com/) using the Illumina Platform PE150 with libraries paired-sequenced (2 x 350 bp). The quality of the raw sequencing data was evaluated using FastQC v0.11.9 [24]. Adapter screening and quality filtering of reads were performed with Trimmomatig 0.39 [25]. De novo genome assemblies were constructed with the SPAdes 3.14 program [26]. Metrics such as N50 and misassembles were obtained with QUAST v5.0.2 [27]. Annotation was performed using the standard operating procedure of IMG Annotation Pipeline v.5.1.0 from the Joint Genome Institute. A second annotation was performed via NCBI using the NCBI Prokaryotic Genome Annotation Pipeline.

### Measurement of genomic relatedness and comparative analysis

The strains were first analyzed using the web server Type (Strain) Genome Server (TYGS) (tygs.dsmz.de), which compares genome sequences with an extensive and continuously updated database of bacterial genome sequences. The genome-to-genome comparison with TYGS uses the formula d4, which is independent of genome length and is robust to incomplete draft genomes [28]. The genome sequences belonging to all type strains of *Cupriavidus* species and *Cupriavidus* sp. strains (i.e., *Cupriavidus* strains not assigned to any described species) were downloaded from the NCBI for genomic comparisons. The digital DNA-DNA hybridization (dDDH) values were estimated using formula 2 of the Genome-to-Genome Distance Calculator (GGDC, http://ggdc.dsmz.de/ggdc.php#9) with 70% as the level of similarity used for species definition [29]. The average nucleotide identity (ANI) values were established using JSpeciesWS online service [30], with values of 95–96% for species definition (29). Protein sequenced-based genome analysis was carried out with the OrthoVenn3 program using the ClusterVenn tool [31] to compare the orthologous clusters of genes among the genomes of strains LEh25^T^, LEh21, and the closest species *C. oxalaticus* Ox1^T^.

### Phylogenomic analysis

A phylogenomic analysis was performed using the up-to-date bacteria core genes (UBCG pipeline) [32] by the maximum likelihood method. Gene support indices (GSI) were used to support the branches. The tree was inferred from 91251 nucleotide positions belonging to 92 core genes. The analysis involved the genome sequence of all *Cupriavidus* type species, the strains LEh25^T^ and LEh21, and the species *Paraburkholderia unamae* MTl-641^T^ and *Paraburkholderia tropica* Ppe8^T^ as outgroups. The tree was displayed with MEGA version 11 [33].

### BOX-PCR

To determine the similarities and differences between the two strains belonging to *Cupriavidus consociatus* sp. nov. and type strains of closely related *Cupriavidus* species, the strains were analyzed using the BOX element (BOXA1), according to a previously described method [34].

### Biochemical and phenotypic tests

Strains LEh25^T^, LEh21, *C. oxalaticus* Ox1^T^, *C. necator* N-1^T,^ and *C. taiwanensis* LMG 19424^T^ were characterized according to different phenotypic features. Colony morphology was determined after two days of culture on LB plates at 30°C. Temperature-dependent growth was evaluated on LB, YM, and MacConkey agar plates at 20, 25, 30, 37, and 42°C after two days. Salt tolerance in modified LB (without NaCl) was examined by adding 0.5, 1, 2, 3, 4, and 5% NaCl; the plates were incubated for five days at 30°C. The effect of pH on bacterial growth was established in LB broth adjusted with the following 1X buffers: a glycine-HCl buffer for ranges of pH 1.0–3.0; an acetate-based buffer for pH 4.0–5.0; a citric acid-phosphate buffer for pH 6.0–7.0; a Tris-HCl buffer for pH 8–9; a glycine-NaOH buffer for pH 10.0–12.0, and a KCl-NaOH buffer for pH 13.0 [35]. The liquid cultures were incubated (120 rpm) for five days at 30°C. Biochemical tests were performed with the VITEK2 System using the VITEK2 GN card according to the manufacturer’s instruction (BIOMÉRIUX).

### Chemotaxonomic characterization

Whole-cell proteins for strains LEh25^T^, LEh21, *C. oxalaticus* Ox1^T^, *C. alkaliphilus* ASC-732^T^, *C. necator* N-1^T^, and *C. taiwanensis* TVV75^T^ were analyzed as described previously by using SDS-PAGE [34]. Briefly, the bacteria were grown in Jain and Patriquin [36] medium with reciprocal shaking (200 rpm) for 15 h at 29°C, and 1.0 mL samples were harvested by centrifugation at 12,300 × *g* for 10 min at 25°C. The pellet was resuspended in 70 μL of 0.125 M Tris-HCl, 4% SDS, 20% glycerol, and 10% mercaptoethanol at pH 6.8. Aliquots of 10 μL were used for SDS-PAGE. Polar lipids were analyzed for strains LEh25^T^, LEh21, and *C. oxalaticus* Ox1^T^. The strains were grown in LB liquid media for 16 h at 29°C, and the polar lipids were extracted following the Bligh and Dyer technique [37]. The chloroform phase was used for lipid analysis by two-dimensional separation on TLC plates [38]. Total polar lipids were imaged by spraying with ANS reagent (8-anilino-1-naphtalenesulfonic acid) [39] and iodine vapor [40]. The polar lipids were identified based on their migration and specific staining. Lipids containing amino groups and glycolipids were determined using the ninhydrin and periodate-Schiff techniques, respectively, as described previously [41,42].

### Ubiquinone analysis *in silico*

The *in silico* analysis of ubiquinones was performed by extracting the amino acid sequences corresponding to UbiA, UbiB, UbiD, UbiE, UbiF, UbiG, UbiH, UbiI, UbiJ, and UbiX from the genome sequences of all type strains of *Cupriavidus* species. The sequences for each protein were individually aligned with Clustal Omega (https://www.ebi.ac.uk/Tools/msa/clustalo/) and then concatenated using the software Mesquite v3.81 (http://www.mesquiteproject.org/?HistoryPanel=open). The phylogenetic analysis used the maximum likelihood method and the aminoacidic model BLOSUM62. The tree was displayed with MEGA version 11 [33], and *Paraburkholderia unamae* MTl-641^T^ and *Paraburkholderia tropica* Pp8^T^ were used as outgroups.

### Nitrogen fixation and nodulation analysis

*Mimosa pudica* seeds were treated with H_2_SO_4_ (95–97%) for 5 min, and *Acacia* sp. and *Leucaena* sp. seeds were treated for 10 min, then washed five times with sterile water. Next, all seeds, including those of *Phaseolus vulgaris* seeds, were disinfected with 10% sodium hypochlorite for 10 min and washed five times with sterile water. The seeds were placed in 15% agar-water plates and incubated at 30°C for 72 h in the dark. The germinated seeds were sown in sterile vermiculite in 200 mL pots and inoculated with 2 mL of a bacterial suspension containing approximately 1 × 10^8^ cells per milliliter. The pots were kept for 45 days in a greenhouse at 30°C with a 14 h light – 10 h dark photoperiod and watered with Fahraeus solution [43]. The experiment was performed with five plants per treatment, with plants grown in individual pots. The treatments were: a) control inoculation with water, b) inoculation with strains LEh25^T^ and LEh21 and c) inoculation with *Paraburkholderia mimosarum* PAS44^T^*, Paraburkholderia tuberum* STM 678^T^, and *Paraburkholderia phymatum* STM 815^T^ as a positive control. After incubation, five nodules were randomly selected from each plant and washed three times with sterile water. The nodule surface was sterilized as described above, and the water from the final rinse was used to verify the disinfection. Finally, the legume nodules were crushed with a plastic pestle in 40 μl of water, and the nodule suspension was inoculated (15 μl) onto plates with YM medium. The plates were incubated at 30°C for 3–5 days. Isolates were identified by amplifying and sequencing the 16S rRNA, as previously analyzed [4], to verify Koch’s postulates. Before the bacterial isolation from nodules, the roots with the nodules were washed with sterile water and placed in 100 mL vials. The vials were sealed with rubber seals, and 5% of the total volume of air was extracted and replaced with acetylene [34]. The roots were then left at room temperature for 8 h. Next, nitrogen fixation was indirectly measured with a Clarus 580 gas chromatographer (PerkinElmer) by the reduction of acetylene to ethylene [34]. In addition, the strains were individually tested for nitrogen fixation by growing the bacteria in 10 mL vials containing 5 mL of semisolid (2.3 g/L) YM medium free of nitrogen for three days. The cotton plug was replaced with a rubber seal, and 10% of the air was replaced with acetylene. The vials were incubated overnight at 30°C, and nitrogen fixation was measured as described above. The free nitrogen fixation included *Paraburkholderia tropica* Ppe8^T^, *Azospirillum brasilense* sp. 7^T^, *Paraburkholderia caballeronis* TNe-862^T^, and *Burkholderia orbicola* TAtl-371^T^.

### Analysis and genetic organization of N-fixation and nodulation genes

The genome sequences of strains LEh25^T^ and LEh21 were explored for nitrogen fixation and nodulation genes. For nitrogen fixation, the *nifHDK* genes were screened in both genomes using the *nifHDK* sequence from *C. taiwanensis* TVV75^T^ that was obtained from the Joint Genome Institute with the GOLD Study ID Gs00111891. A phylogenetic analysis of the amino acid NifH sequence was performed with several nitrogen-fixing bacteria, primarily from the genera *Paraburkholderia*, *Cupriavidus*, and *Trinickia,* and the species *Rhizobium etli* CFN42^T^ and *Bradyrhizobium japonicum* ATCC 10324^T^ were used as the outgroups. The phylogenetic analysis used the maximum likelihood method and the amino acid substitution model BLOSUM62. The tree was displayed with MEGA version 11 [33]. Bootstrap analysis was performed with 1000 replications. For the analysis of nodulation genes, the organization of *nodBDCIJHASU* genes was searched in both genomes using the genes from *C. taiwanensis* TVV75^T^, also gathered from the JGI database. A phylogenetic analysis of the amino acid NodC sequence was carried out with the strains of the novel species and other strains from species of *Cupriavidus*, *Paraburkholderia*, *Trinickia*, *Rhizobium,* and *Mesorhizobium*. The phylogenetic tree was performed similarly to the one for NifH.

## Results and discussion

### Phylogenetic analysis based on the 16S rRNA gene sequence

**Fig 1:**
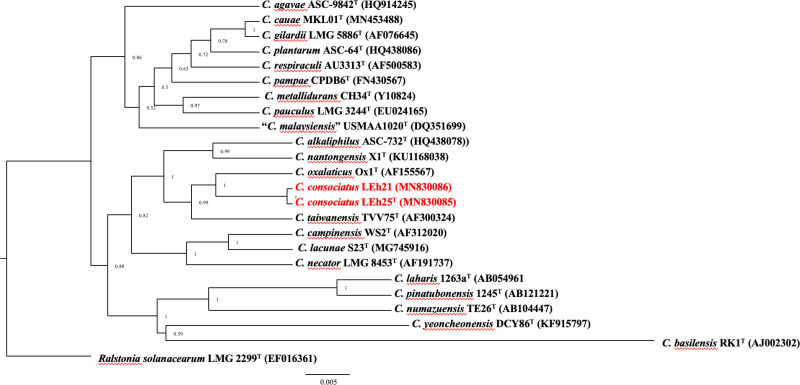
Phylogenetic analysis of *Cupriavidus* species is based on a comparison of 16S rRNA gene sequences. The study was performed with 1550 nucleotides using the Bayesian inference method with the Beast software v 2.5. The analysis included the GTR + I + G model of nucleotide substitution. The novel species *Cupriavidus consociatus* sp. nov. is shown in red. The numbers in parentheses correspond to the accession numbers in the NCBI database. The numbers at branch points indicate bootstrap support. The bar represents substitutions per nucleotide position. *Ralstonia solanacearum* LMG 2299^T^ was used as the outgroup.

The newly obtained 16S rRNA gene sequences from both strains LEh25^T^ and LEh21 were identical. The sequences were compared to those of all type strains of *Cupriavidus* species; the results showed 98.5% similarity to *C. oxalaticus* Ox1^T^. For bacteria, 98.7% is the accepted threshold for species delineation [44]. Therefore, the results indicate that strains LEh25^T^ and LEh21 belong to a new species of *Cupriavidus*. Moreover, the 16S rRNA sequences from strains LEh25^T^ and LEh21 were identical to the sequence obtained from the genome. The phylogenetic analysis grouped both strains with *C. oxalaticus* Ox1^T^ and the neighboring *C. taiwanensis* TVV75^T^ (Fig 1). The analysis corroborated the identification of the strains LEh25^T^ and LEh21 as members of the genus *Cupriavidus*.

### General features of the genome

The genome sequences of strains LEh25^T^ and LEh21 were assembled in 237 (total length 8,337,954 bp) and 191 (8,247,320 bp) contigs, respectively. The G + C content was 65.19% for strain LEh25^T^ and 65.26% for strain LEh21. The content of ribosomal genes was similar between the two strains of the new species, although the number was low. This may have been due to the genome assembly since different strains from *C. oxalaticus*, the closest species, also contained a variable number of ribosomal genes, from zero to nine (data not shown). More information concerning the genomes for strains LEh25^T^ and LEh21 is summarized in the S1 Table.

### Measurements of genomic relatedness

The evaluation of the genome sequence from the two *Cupriavidus* strains on the TYGS website showed that the strains represented a new genomic species closely related to *C. oxalaticus* Ox1^T^ (~ 50% similarity). Both strains belonged to the same genomic species (99.99% similarity). The results of the analysis with dDDH and ANI revealed that the similarity to *C. oxalaticus* Ox1^T^ was 50.1–50.2% and 93.22–93.23% for strains LEh25^T^ and LEh21, respectively (Table 1) values lower than the criteria for the species cut-off. Moreover, the comparison with *Cupriavidus* sp. (i.e., *Cupriavidus* strains not assigned to any described species) also showed values lower than the cut-off.

**Table 1:** Comparative genomics between the novel species *Cupriavidus consociatus* sp. nov. and other type strains of *Cupriavidus* species.

*Cupriavidus* species	ANI		dDDH	
**LEh25** ^**T**^	**LEh21**	**LEh25** ^**T**^	**LEh21**
*C. oxalaticus* Ox1^T^	90.16	90.25	50.1	50.2
*C. alkaliphilus* ASC-732^T^	83.99	84.06	31.5	31.5
*“C. neocaledonicus”* STM 6070^T^	84.03	84.06	31.7	31.7
*C. taiwanensis* LMG 19424^T^	83.86	83.91	31.7	31.7
*C. nantongensis* X1^T^	83.49	83.62	31.3	31.3
*C. lacunae* S23^T^	84.04	84.03	31.4	31.4
*C. necator* N-1^T^	84.53	84.61	38.7	38.7
*C. laharis* LMG 23992^T^	80.35	80.42	26.6	26.6
*C. pinatubonensis* LMG 23994^T^	80.53	80.61	26.0	25.9
*C. campinensis* LMG 19282^T^	78.5	78.61	24.5	24.5
*C. cauae* MKL-01^T^	78.37	78.56	25.5	25.5
*C. respiraculi* LMG 21510^T^	77.87	78.07	24.1	24.1
*C. agavae* ASC-9842^T^	78.13	78.22	24.2	24.2
*C. gilardii* CCUG 38401^T^	78.13	78.22	24.8	24.8
*“C. malaysiensis”* USMAA1020^T^	78.53	78.66	24.6	24.6
*C. basilensis* CCUG 49340^T^	78.19	78.15	24.3	24.2
*C. pauculus* CCUG 12507^T^	77.42	78.15	23.7	23.7
*C. metallidurans* CH 34^T^	77.37	77.84	23.6	23.6
*C. plantarum* LMG 26296^T^	77.14	77.23	23.9	23.9
*C. pampae* LMG 32289^T^	76.92	77.00	23.8	23.8

The ANI and dDDH values between the strains LEh25^T^ and LEh21 are 99.99% and 99.90%, respectively.

**Fig 2:**
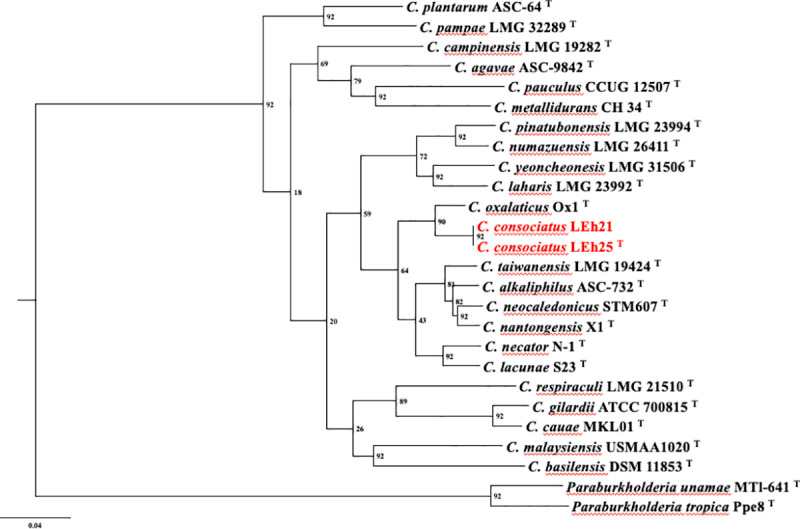
Phylogenomic analysis of *Cupriavidus* species using the up-to-date bacterial concatenated alignment of 92 core genes (UBCG). A total of 91251 nucleotide positions were used. The phylogenomic tree was inferred using the maximum likelihood method. Gene support indices (GSI) are given at the branching points. Bar = 0.04 substitutions per position. *Paraburkholderia unamae* MTl-641^T^ and *Paraburkholderia tropica* Ppe8^T^ were used as the outgroups.

The phylogenomic analysis using the genome sequence from all type strains of *Cupriavidus* species indicated that the novel genomic species formed a single cluster near *C. oxalaticus* Ox1^T^ (Fig 2), demonstrating that strains LEh25^T^ and LEh21 belong to a new genomic species within the genus *Cupriavidus.*

### Comparative genomics

**Fig 3:**
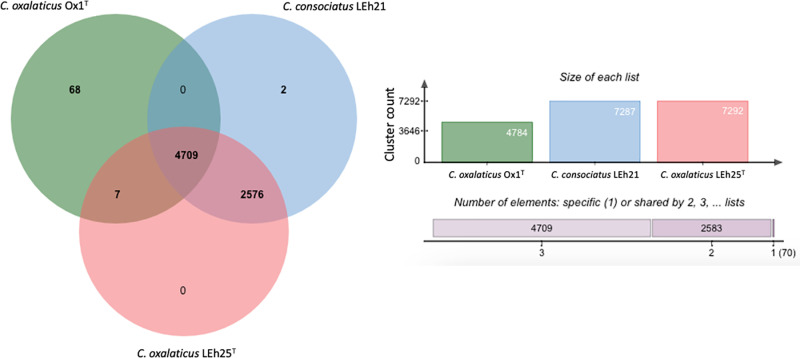
Venn diagram of the common and unique orthologous genes among *Cupriavidus consociatus* sp. nov. LEh25^T^, and LEh21, with *Cupriavidus oxalaticus* Ox1^T^, using the software OrthoVenn2 [45].

The whole-genome orthologous gene comparison (Fig 3) showed that strains LEh25^T^, LEh21, and Ox1^T^ formed 7362 clusters of genes, 7287 that were typical for the strains of the novel species, a value that was higher than the 4709 between strain LEh25^T^ and *C. oxalaticus* Ox1^T^ or 4716 between strain LEh21 and *C. oxalaticus* Ox1^T^. This illustrated the differences between the novel species and its closest relative. There were 1113 singletons or proteins not in any cluster, with 1010 belonging only to *C. oxalaticus* Ox1^T^. The three strains shared 4709 clusters, with these being associated with molecular function (126), hydrolase activity (92), and oxidoreductase activity (90).

### BOX-PCR

**Fig 4:**
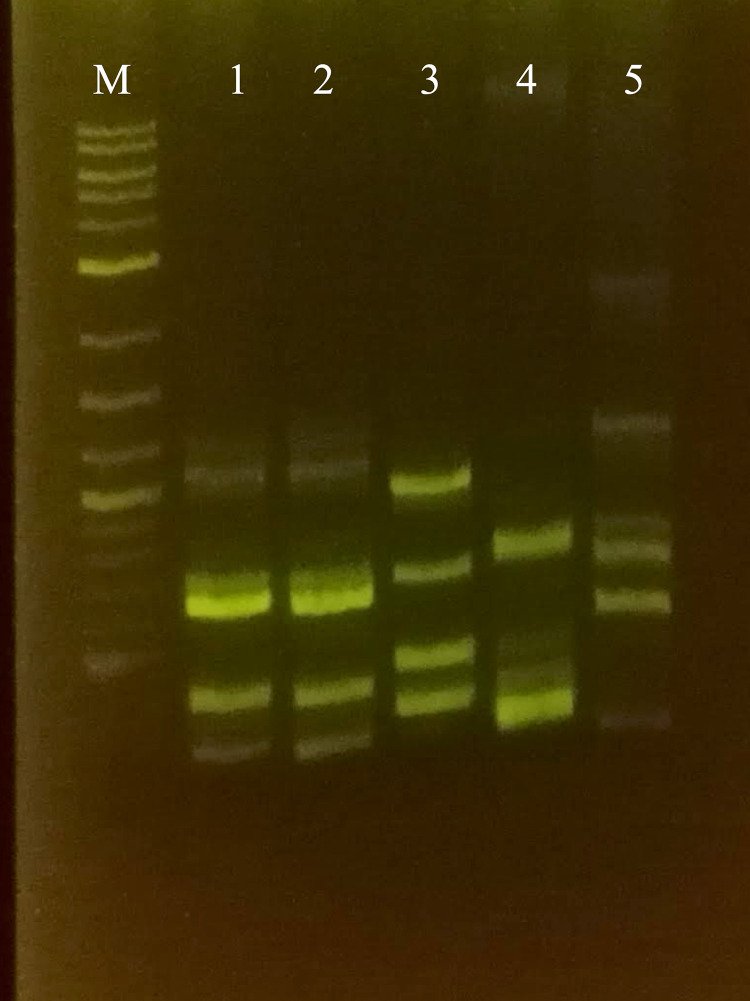
BOXA1 patterns among *Cupriavidus consociatus* sp. nov. and several type strains of *Cupriavidus* species. M, molecular marker. 1, *Cupriavidus consociatus* sp. nov. LEh25^T^. 2, *Cupriavidus consociatus* sp. nov. LEh21. 3, *Cupriavidus oxalaticus* Ox1^T^. 4, *Cupriavidus taiwanensis* LMG 19424^T^. 5, *Cupriavidus necator* N-1^T^.

Fingerprinting obtained with BOX-PCR can help to discriminate bacterial strains at the species level [46]. The comparative analysis showed identical patterns between strains LEh25^T^ and LEh21 and completely different patterns with the type strains of *C. oxalaticus*, *C. taiwanensis*, and *C. necator* (Fig 4).

### Biochemical and phenotypic analysis

The bacterial cells stained Gram-negative. The colonies grown in LB after 48 h were circular, convex, and whitish-colored. The strains grew in LB, YM, and MacConkey agar at 20, 25, 30, 37, and 42°C after 48 h. The strains were able to grow in LB up to 2% NaCl. The pH range for growth was 5–9. The alkalinization of L-lactate and succinate was positive. Both had activity of gamma-glutamyl-transferase, L-proline-arylamidase, and tyrosine arylamidase. The strains assimilated sodium citrate, malonate, L-malate, Ellman, and L-lactate. The complete results from biochemical and phenotypic analysis are displayed in S2 Table, and differential features with the closest species are listed in Table 2.

**Table 2:** Differential phenotypic features between *Cupriavidus consociatus* sp. nov. and closest and relevant *Cupriavidus* type species.

Phenotypic feature	*C. consociatus* sp. nov. LEh25^T^	*C. consociatus* sp. nov. LEh21	*C. oxalaticus* Ox1^T^	*C. necator* N-1^T^	*C. taiwanensis* LMG 19424^T^
Isolation source	*Arachis* sp. nodules	*Leucaena* sp. nodules	Alimentary tract earthworm	Soil	*Mimosa pudica* nodules
Location source	Mexico	Mexico	India	USA	Taiwan
NaCl tolerance range (%)	0 − 2	0 − 2	0 − 2	0 − 2	0 − 3
Growth pH range	5 − 9	6 − 9	8 − 9	6 − 9	6 − 9
Nodulation activity	+	+	−	−	+
Free nitrogen fixation	+	+	−	−	ND
Activity of: L-Pyrrolydonyl-arylamidase Glutamyl arylamidase pNA Gamma-glutamyl-transferase Urease Phosphatase Glycine arylamidase	− − + − − −	− − + − − −	− − − − + −	+ + + − + −	+ + + + + +
Assimilation of: Malonate L-malate L-lactate	+ + +	+ + +	− − −	− + +	− + +

+, positive test. − , negative test. ND, Not determined

### Chemotaxonomic characterization

**Fig 5:**
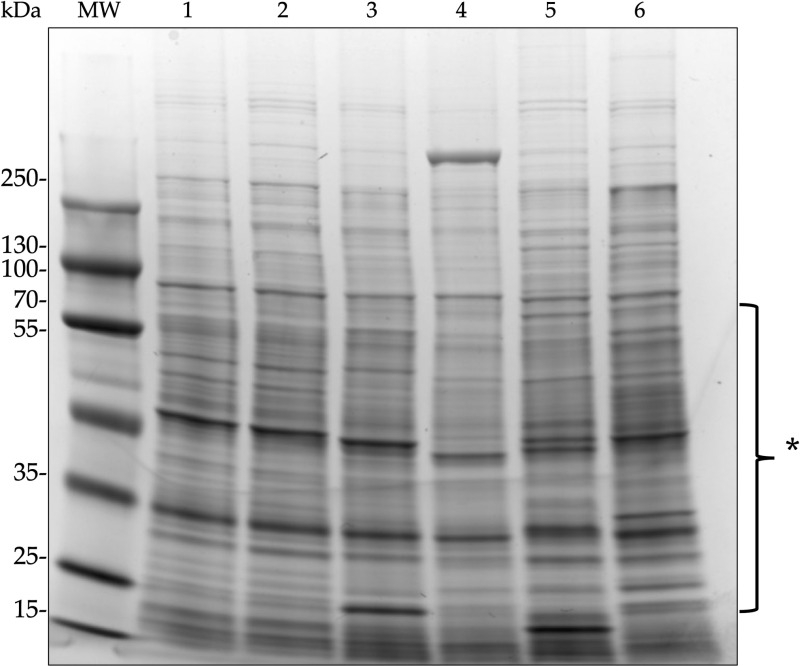
Protein electropherograms (SDS-PAGE) of *Cupriavidus consociatus* sp. nov. and closely related and relevant type strains of *Cupriavidus* species. Strains: 1, LEh25^T^; 2, LEh21; 3, *C. oxalaticus* Ox1^T^; 4, *C. taiwanensis* TVV75^T^; 5, *C. alkaliphilus* ASC-732^T^; 6, *C. necator* N1^T^. MW, PageRuler molecular marker. The asterisk marks differences between strains LEh25^T^ and LEh21, with *C. oxalaticus* Ox1^T^.

Whole-cell protein patterns were analyzed in strains LEh25^T^, LEh21, *C. oxalaticus* Ox1^T^, *C. alkaliphilus* ASC-732^T^, *C. necator* N-1^T^, and *C. taiwanensis* LMG 19424^T^. The analysis of the total cellular protein electrophoretic patterns provides discriminative information at or below the species level [47]. The protein patterns in the strains LEh25^T^ and LEh21 were identical but different from those of the other type strains of *Cupriavidus* species tested (Fig 5), thus illustrating the similarity in the novel species and the differences with other type strains of *Cupriavidus* species.

**Fig 6:**
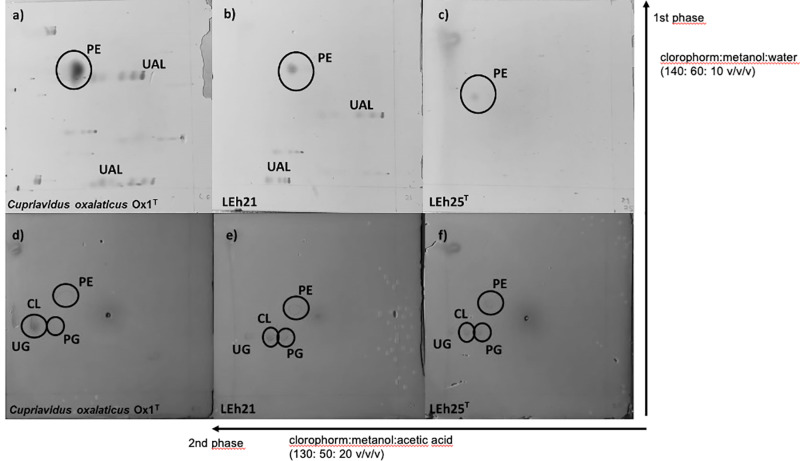
Membrane lipid profiles of *Cupriavidus consociatus* sp. nov. and *Cupriavidus oxalaticus* Ox1^T^. The first TLC was stained with ninhydrin (a, b, and c), presenting PE (phosphatidylethanolamine) and UAL (an unknown amino lipid). The second TLC was stained with Shiff’s reagent (d, e, and f), showing PG (phosphatidylglycerol), CL (cardiolipin), and UG (an unknown glycolipid).

The analysis of polar lipids was performed four times in independent experiments. In each of these experiments, strain LEh25^T^ resulted in faint spots when stained with ninhydrin, although phosphatidylethanolamine (PE) was identified (Fig 6). This was a difference between strains LEh25^T^ and LEh21; the latter, besides PE, also contained two unknown amino lipids. Other polar lipids consisted of phosphatidylglycerol (PG), cardiolipin (CL), and an unknown glycolipid (UG).

### Ubiquinones analysis *in silico*

**Fig 7:**
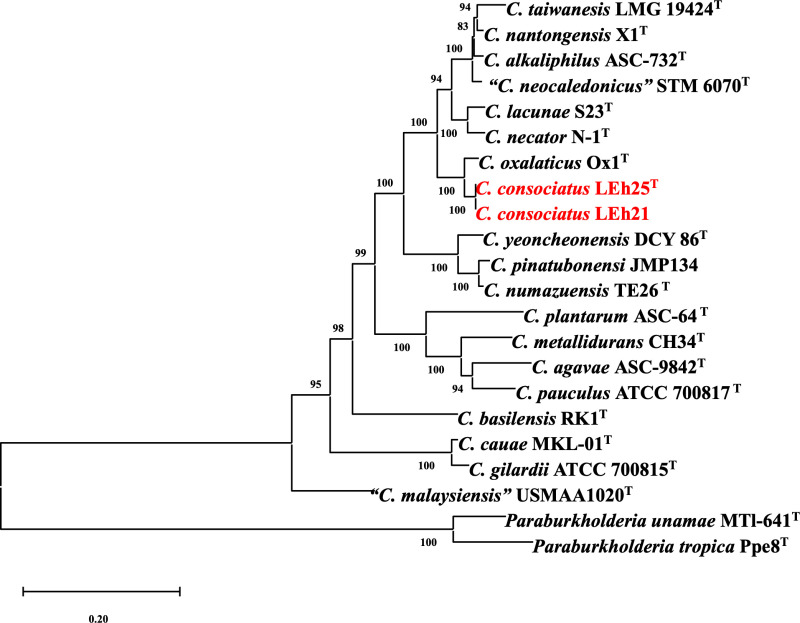
Phylogenetic analysis of concatenated ubiquinone amino acid sequences from type strains of *Cupriavidus* species. The phylogenetic analysis used the maximum likelihood method and the amino acid substitution model BLOSUM62. The numbers at the branch points represent bootstrap support generated from 1000 replications. Bar = 0.2 nucleotide substitutions per position.

The amino acid sequences corresponding to UbiA, UbiB, UbiD, UbiE, UbiF, UbiG, UbiH, UbiI, UbiJ, and UbiX from all type strains of *Cupriavidus* species were used in a phylogenetic analysis. The sequences were concatenated, producing a total of 3914 positions. The phylogenetic analysis showed that the novel species grouped with *C. oxalaticus* Ox1^T^ (Fig 7), consistent with the genome sequence comparison analysis. The major isoprenoid quinone reported in nine *Cupriavidus* species is ubiquinone Q-8. Thus, the association of the novel species with all type strains of *Cupriavidus* species in the phylogenetic analysis of ubiquinone amino acid sequences suggests that the novel species may contain ubiquinone Q-8.

### Nitrogen fixation and nodulation analysis *in vitro*, *in vivo* and *in silico*

**Fig 8:**
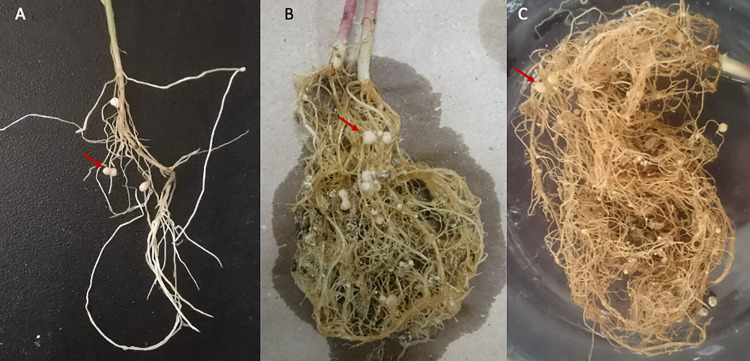
Nodulation activity of *Cupriavidus consociatus* sp. nov. A) *Leucaena* sp. and B) *Phaseolus vulgaris* by *Cupriavidus consociatus* sp. nov. LEh25^T^. C) *P. vulgaris* by *Cupriavidus consociatus* sp. nov. LEh21. The arrows indicate white, ineffective nodules.

The ability of the novel species to fix nitrogen in a nitrogen-free culture medium was assessed, and the results were negative for both the novel species and *B. orbicola*. The positive controls *P. tropica*, *A. brasilense* and *P. caballeronis* fixed nitrogen in the semisolid medium. The capacity to fix nitrogen in culture media has not been thoroughly explored in the genus *Cupriavidus*, for example, *C. necator* was unable to grow in Burk’s N-free medium supplemented with glucose or sucrose, and it displayed limited growth with fructose that was not sustained after several passes in an N-free medium [48]. An experimental analysis employing the inoculation of the novel species to legume plants indicated that the bacteria could associate with *P. vulgaris* and *Leucaena* sp. in the case of strain LEh25^T^ and only *P. vulgaris* by strain LEh21 (Fig 8). In both cases, the nodules were white and ineffective regarding nitrogen fixation. Neither *M. pudica* nor *Acacia* sp. showed nodules when the plants were inoculated with the strains LEh25^T^ and LEh21. Nodulation of *P. vulgaris* and *M. pudica* was carried out by the control species *P. mimosarum*, *P. tuberum* and *P. phymatum*.

**Fig 9:**
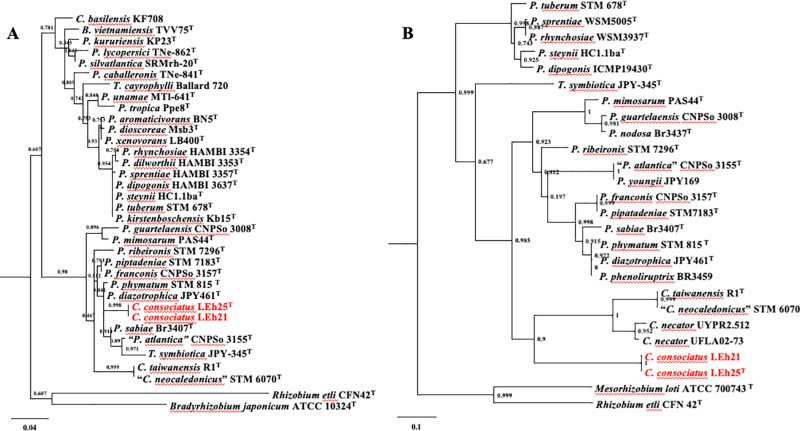
Phylogenetic analysis of NifH and NodD amino acid sequences from *Cupriavidus consociatus* sp. nov. and other nitrogen-fixing and nodulating bacteria **(A)** **NifH corresponds to nitrogen fixation activity.** (B) NodC corresponds to nodulation activity. The phylogenetic analysis used the maximum likelihood method and the amino acid substitution model BLOSUM62. The numbers at branch points represent bootstrap support generated from 1000 replications. Bar = number of nucleotide substitutions per position.

The search for nitrogen fixation genes in the genome (genes *nifHDK*) of strains LEh25^T^ and LEh21 found a single copy with an identical arrangement to those of other *Cupriavidus* and *Paraburkholderia* strains. The NifHDK amino acid sequence identity between strains LEh25^T^ and LEh21 was 100% and ranged from 95 to 100% with other *Cupriavidus* and *Paraburkholderia* species. The phylogenetic analysis using NifH amino acid sequences with mostly type strains indicated the position of the novel species among *Paraburkholderia*, with *P. diazotrophica* JPY461^T^ as the closest species (95% identity). The *nod* genes *nodDCIJHASU* were identically organized between strains LE25^T^ and LEh21 (100% identity) as with other *Paraburkholderia* and *Cupriavidus* strains. However, NodB was absent in the genome of strains LEh25^T^ and LEh21. NodB is an oligosaccharide deacetylase that plays a central role in the Nod-factor biosynthesis and the symbiotic nodulation in plants. Possibly, this could be why neither strain could form nitrogen-fixing nodules. The phylogenetic analysis of NodC sequences showed that the novel species formed a closed group with other type strains of *Cupriavidus* species, different from *Paraburkholderia* and *Trinickia* strains (Fig 9).

### Conclusions

In this study, two *Cupriavidus* strains, LEh25^T^ and LEh21, were isolated from two legume plants growing in the wild in the south of Mexico. A comparative genomic analysis and phenotypic exploration revealed that both strains belonged to a novel species. Thus, we conclude that the two strains should be assigned to a new species in the genus *Cupriavidus*, and we propose the name *Cupriavidus consociatus* sp. nov. with LEh25^T^ (=TSC-314^T^ = CDBB B-2085^T^) as the type strain.

### Description of *Cupriavidus consociatus* sp. nov.

*Cupriavidus consociatus* (con.so.ci.a’tus. L. masc. part. adj. *consociatus*, associated with, in this case, the root nodules of legume plants).

The cells are Gram-staining-negative, aerobic. The colonies growing in LB are circular, convex, and whitish-colored. The bacteria are able to grow in LB, YM, and MacConkey agar at 20, 25, 30, 37, and 42°C, in up to 2% NaCl, and in the range of pH values of 5–9. There is no production of H_2_S, but there is alkalinization of L-lactate and succinate. There are activities of gamma-glutamyl-transferase, L-proline-arylamidase, and tyrosine arylamidase, but no activity of Ala-Fe-Pro-arylamidase, L-pyrrolydonyl-arylamidase, beta-galactosidase, beta-N-acetyl-glucosaminidase, glutamyl arylamidase pNA, beta-glucosidase, beta-xylosidase, beta-alanine arylamidase pNA, lipase, palatinose, urease, alpha-glucosidase, beta-N-acetyl-galactominidase, alpha-galactosidase, phosphatase, glycine arylamidase, ornithine decarboxylase, lysine decarboxylase, beta-glucuronidase, or Glu-Gly-Arg-arylamidase. The species can assimilate sodium citrate, malonate, L-malate, Ellman, and L-lactate but not adonitol, L-arabitol, D-cellobiose, D-glucose, D-maltose, D-mannitol, D-mannose, D-sorbitol, saccharose, D-tagatose, D-trehalose, 5-keto-D-gluconate, L-histidine, and coumarate. Test for glucose fermentation and O/129 resistance were negative. Total polar lipids consisted of phosphatidylethanolamine (PE), an unknown amino lipid (UAL), phosphatidylglycerol (PG), cardiolipin (CL), and an unknown glycolipid (UG). Both strains have *nif* and *nod* genes but were unable to fix nitrogen and strain LEh25^T^ produced ineffective nodules.

The type strain LEh25^T^=TSD-314^T^ = CDBB B-2085^T^ was isolated from root nodules from *Arachis* sp. growing in a cacao field in Chiapas, México.

### Nucleotide sequence accession number

The GenBank accession numbers for the 16S rRNA gene sequences of strains LEh25^T^ and LEh21 are MN830085 and MN830086, respectively. The GenBank accession numbers for the genome sequences of strains LEh25^T^ and LEh21 are JAGIQA000000000 and JASMMW000000000.1, respectively.

## Supporting information

S1 Table Genomic features of *Cupriavidus consociatus* sp. nov. LEh25^T^ and LEh21. (DOCX)

S2 Table Phenotypic features between *Cupriavidu consociatus* sp. nov. and close and relevant *Cupriavidus* type species. (DOCX)

S3 Table Genome and ubiquinone protein accession numbers. (XLSX)

S4 Table Genome and NifH protein accession numbers. (XLSX)

S5 Table Genome and NodC protein accession numbers. (XLSX)
