# Supplementary material for: A new nodule-associated bacterium, Cupriavidus consociatus sp. nov. Isolated from the root nodules of Leucaena sp. and Arachis sp. growing in a cacao field in Chiapas, Mexico
Source: PLoS One. 2025 May 27;20(5):e0324390. doi: 10.1371/journal.pone.0324390 (PMC12180409; doi:10.1371/journal.pone.0324390)
Supplement: S1 Table — (DOCX) [file pone.0324390.s001.docx]

**S1 Table. Genomic features of *Cupriavidus consociatus* sp. nov. LEh25^T^ and LEh21.**

| **Genomic features** | **LEh25^T^** | **LEh21** |
| --- | --- | --- |
| Genome length (bp) | 8,337,954 | 8,247,320 |
| Number of contigs | 237 | 191 |
| G + C content (%) | 65.19 | 65.26 |
| CRISPR Count | 2 | 2 |
| CDS | 7,721 | 7,633 |
| RNA genes | 80 | 82 |
| rRNA genes  5S rRNA  16S rRNA  23S rRNA | 9  2  1  3 | 8  1  2  5 |
| tRNA genes | 67 | 67 |
| Protein coding genes with function prediction  without function prediction | 6,346  1,375 | 6,308  1,325 |
| Protein coding genes with enzymes | 2,099 | 2,123 |
| Protein coding genes connected to KEGG pathways  not connected to KEGG pathways | 2,324  5397 | 2,339  5,294 |
| Protein coding genes connected to KEGG Orthology (KO)  not connected to KO | 3,814  3,907 | 3,831  3,802 |
| Protein coding genes connected to MetaCyc pathways  not connected to MetaCyc pathways | 1,841  5,880 | 1,860  5,773 |
| Protein coding genes with COGs  with Pfam  with TIGRfam  with SMART  with SUPERFam  with CATH FunFam  in internal clusters | 6,274  6,450  1,913  1,451  6,456  5,293  3,283 | 6,229  6,400  1,918  1,440  6,414  5,265  3,233 |
| Protein coding genes coding signal peptides | 800 | 800 |
| Protein coding genes coding transmembrane proteins | 1,638 | 1,643 |
| COG clusters | 2,179 | 2,180 |
| Pfam clusters | 2,715 | 2,713 |
| TIGRfam clusters | 1,310 | 1,310 |
| Completeness (%) | 97.89 | 99.59 |
| Contamination (%) | 3.61 | 3.32 |
