# Supplementary material for: A new nodule-associated bacterium, Cupriavidus consociatus sp. nov. Isolated from the root nodules of Leucaena sp. and Arachis sp. growing in a cacao field in Chiapas, Mexico
Source: PLoS One. 2025 May 27;20(5):e0324390. doi: 10.1371/journal.pone.0324390 (PMC12180409; doi:10.1371/journal.pone.0324390)
Supplement: S2 Table — (DOCX) [file pone.0324390.s002.docx]

**S2 Table. Phenotypic features between *Cupriavidus consociatus* sp. nov. and close and relevant type strains of *Cupriavidus* species.**

| **Phenotypic feature** | ***C. consociatus* sp. nov.**  **LEh25^T^** | ***C. consociatus* sp. nov.**  **LEh21** | ***C. oxalaticus***  **Ox1^T^** | ***C. alkaliphilus***  **ASC-732^T^** | ***C. necator***  **N-1^T^** | ***C. taiwanensis***  **LMG 19424^T^** |
| --- | --- | --- | --- | --- | --- | --- |
| **Isolation source** | ***Leucaena* sp.**  **nodules** | ***Arachis* sp.**  **nodules** | **Alimentary tract**  **earthworm** | ***Agave* sp.**  **rhizosphere** | **Soil** | ***Mimosa pudica***  **nodules** |
| **Location source** | **Chiapas, Mexico** | **Chiapas, Mexico** | **India** | **Tamaulipas, Mexico** | **United States** | **Taiwan** |
| **Gram stain** | **−** | **−** | **−** | **−** | **−** | **−** |
| **Growth on LB agar at:** | | | | | | |
| **20 °C**  **25 °C**  **30 °C** | **+**  **+**  **+** | **+**  **+**  **+** | **+**  **+**  **+** | **nd**  **nd**  **+** | **+**  **+**  **+** | **+**  **+**  **+** |
| **37 °C** | **+** | **+** | **+** | **+** | **+** | **+** |
| **42 °C** | **+** | **+** | **+** | **+** | **+** | **+** |
| **Growth on YM agar at:** | | | | | | |
| **30 °C** | **+** | **+** | **+** | **+** | **+** | **+** |
| **37 °C** | **+** | **+** | **+** | **+** | **+** | **+** |
| **42 °C** | **+** | **+** | **+** | **+** | **+** | **+** |
| **Growth on MacConkey agar at:** | | | | | | |
| **30 °C** | **+** | **+** | **+** | **+** | **+** | **+** |
| **37 °C** | **+** | **+** | **+** | **+** | **+** | **+** |
| **42 °C** | **+** | **+** | **+** | **+** | **+** | **+** |
| **Growth on LB agar + NaCl (%):** | | | | | | |
| **0.0** | **+** | **+** | **+** | **+** | **+** | **+** |
| **0.5** | **+** | **+** | **+** | **+** | **+** | **+** |
| **1.0** | **+** | **+** | **+** | **+** | **+** | **+** |
| **2.0** | **+** | **+** | **+** | **+** | **+** | **+** |
| **3.0** | **−** | **−** | **−** | **+** | **−** | **+** |
| **4.0** | **−** | **−** | **−** | **+** | **−** | **−** |
| **5.0** | **−** | **−** | **−** | **−** | **−** | **−** |
| **Growth at pH values:** | | | | | | |
| **1** | **−** | **−** | **−** | **−** | **−** | **−** |
| **2** | **−** | **−** | **−** | **−** | **−** | **−** |
| **3** | **−** | **−** | **−** | **−** | **−** | **−** |
| **4** | **−** | **−** | **−** | **−** | **−** | **−** |
| **5** | **+** | **−** | **−** | **+** | **−** | **−** |
| **6** | **+** | **+** | **−** | **+** | **+** | **+** |
| **7** | **+** | **+** | **−** | **+** | **+** | **+** |
| **8** | **+** | **+** | **+** | **+** | **+** | **+** |
| **9** | **+** | **+** | **+** | **+** | **+** | **+** |
| **10** | **−** | **−** | **−** | **−** | **−** | **−** |
| **11** | **−** | **−** | **−** | **−** | **−** | **−** |
| **12** | **−** | **−** | **−** | **−** | **−** | **−** |
| **13** | **−** | **−** | **−** | **−** | **−** | **−** |
| **H_2_S production** | **−** | **−** | **−** |  | **−** | **−** |
| **Alkalinization of:** | | | | | | |
| **L-lactate** | **+** | **+** | **+** |  | **+** | **+** |
| **Succinate** | **+** | **+** | **+** |  | **+** | **+** |
| **Activity of:** | | | | | | |
| **Ala-Fe-Pro-arylamidase** | **−** | **−** | **−** |  | **−** | **−** |
| **L-Pyrrolydonyl-arylamidase** | **−** | **−** | **−** |  | **+** | **+** |
| **Beta-galactosidase** | **−** | **−** | **−** |  | **−** | **−** |
| **Beta-N-acetyl-glucosaminidase** | **−** | **−** | **−** |  | **−** | **−** |
| **Glutamyl arylamidase pNA** | **−** | **−** | **−** |  | **+** | **+** |
| **Gamma-glutamyl-transferase** | **+** | **+** | **−** |  | **+** | **+** |
| **Beta-glucosidase** | **−** | **−** | **−** |  | **−** | **−** |
| **Beta-xylosidase** | **−** | **−** | **−** |  | **−** | **−** |
| **Beta-alanine arylamidase pNA** | **−** | **−** | **−** |  | **­−** | **−** |
| **L-proline-arylamidase** | **+** | **+** | **+** |  | **+** | **+** |
| **Lipase** | **−** | **−** | **−** |  | **−** | **−** |
| **Palatinose** | **−** | **−** | **−** |  | **−** | **−** |
| **Tyrosine arylamidase** | **+** | **+** | **+** |  | **+** | **+** |
| **Urease** | **−** | **−** | **−** |  | **−** | **+** |
| **Alpha-glucosidase** | **−** | **−** | **−** |  | **−** | **−** |
| **Beta-N-acetyl-galactominidase** | **−** | **−** | **−** |  | **−** | **−** |
| **Alpha-galactosidase** | **−** | **−** | **−** |  | **−** | **−** |
| **Phophatase** | **−** | **−** | **+** |  | **+** | **+** |
| **Glycine arylamidase** | **−** | **−** | **−** |  | **−** | **+** |
| **Ornithine decarboxylase** | **−** | **−** | **−** |  | **−** | **−** |
| **Lysine decarboxilase** | **−** | **−** | **−** |  | **−** | **−** |
| **Beta-glucuronidase** | **−** | **−** | **−** |  | **−** | **−** |
| **Glu-Gly-Arg-arylamidase** | **−** | **−** | **−** |  | **−** | **−** |
| **Assimilation of:** | | | | | | |
| **Adonitol** | **−** | **−** | **−** |  | **−** | **−** |
| **L-arabitol** | **−** | **−** | **−** |  | **−** | **−** |
| **D-cellobiose** | **−** | **−** | **−** |  | **−** | **−** |
| **D-glucose** | **−** | **−** | **−** |  | **−** | **−** |
| **D-maltose** | **−** | **−** | **−** |  | **−** | **−** |
| **D-mannitol** | **−** | **−** | **−** |  | **−** | **−** |
| **D-mannose** | **−** | **−** | **−** |  | **−** | **−** |
| **D-sorbitol** | **−** | **−** | **−** |  | **−** | **−** |
| **Saccharose** | **−** | **−** | **−** |  | **­−** | **−** |
| **D-tagatose** | **−** | **−** | **−** |  | **−** | **−** |
| **D-trehalose** | **−** | **−** | **−** |  | **−** | **−** |
| **Citrate (sodium)** | **+** | **+** | **+** |  | **+** | **+** |
| **Malonate** | **+** | **+** | **−** |  | **−** | **−** |
| **5-keto-D-gluconate** | **−** | **−** | **−** |  | **−** | **−** |
| **L-histidine** | **−** | **−** | **−** |  | **−** | **−** |
| **Coumarate** | **−** | **−** | **−** |  | **−** | **­−** |
| **L-malate** | **+** | **+** | **−** |  | **+** | **+** |
| **Ellman** | **+** | **+** | **+** |  | **+** | **+** |
| **L-lactacte** | **+** | **+** | **−** |  | **+** | **+** |
| **Glucose fermentation** | **−** | **−** | **−** |  | **−** | **−** |
| **O/129 resistance** | **−** | **−** | **−** |  | **−** | **−** |
